# Development of 2, 7-Diamino-1, 8-Naphthyridine (DANP) Anchored Hairpin Primers for RT-PCR Detection of Chikungunya Virus Infection

**DOI:** 10.1371/journal.pntd.0004887

**Published:** 2016-08-29

**Authors:** Huixin Chen, Mariya Parimelalagan, Fumie Takei, Hapuarachchige Chanditha Hapuarachchi, Evelyn Siew-Chuan Koay, Lee Ching Ng, Phui San Ho, Kazuhiko Nakatani, Justin Jang Hann Chu

**Affiliations:** 1 Laboratory of Molecular RNA Virology and Antiviral Strategies, Department of Microbiology and Immunology, Yong Loo Lin School of Medicine, National University Health System, National University of Singapore, Singapore; 2 School of Applied Science, Republic Polytechnic, Singapore; 3 The National Defense Medical College, Tokorozawa, Saitama, Japan; 4 Environmental Health Institute, National Environment Agency, Singapore; 5 Molecular Diagnosis Centre, Department of Laboratory Medicine and Pathology, Yong Loo Lin School of Medicine, National University Health System, Level 3, National University Hospital Main Building, Singapore; 6 The Institute of Scientific and Industrial Research, Osaka University, Ibaraki, Japan; Naval Medical Research Center, UNITED STATES

## Abstract

A molecular diagnostic platform with DANP-anchored hairpin primer was developed and evaluated for the rapid and cost-effective detection of Chikungunya virus (CHIKV) with high sensitivity and specificity. The molecule 2, 7-diamino-1, 8-naphthyridine (DANP) binds to a cytosine-bulge and emits fluorescence at 450 nm when it is excited by 400 nm light. Thus, by measuring the decline in fluorescence emitted from DANP—primer complexes after PCR reaction, we could monitor the PCR progress. By adapting this property of DANP, we have previously developed the first generation DANP-coupled hairpin RT-PCR assay. In the current study, we improved the assay performance by conjugating the DANP molecule covalently onto the hairpin primer to fix the DANP/primer ratio at 1:1; and adjusting the excitation emission wavelength to 365/430 nm to minimize the background signal and a ‘turn-on’ system is achieved. After optimizing the PCR cycle number to 30, we not only shortened the total assay turnaround time to 60 minutes, but also further reduced the background fluorescence. The detection limit of our assay was 0.001 PFU per reaction. The DANP-anchored hairpin primer, targeting *nsP2* gene of CHIKV genome, is highly specific to CHIKV, having no cross-reactivity to a panel of other RNA viruses tested. In conclusion, we report here a molecular diagnostic assay that is sensitive, specific, rapid and cost effective for CHIKV detection and can be performed where no real time PCR instrumentation is required. Our results from patient samples indicated 93.62% sensitivity and 100% specificity of this method, ensuring that it can be a useful tool for rapid detection of CHIKV for outbreaks in many parts of the world.

## Author Summary

Chikungunya has reemerged as an important mosquito-borne infection with global health significance. Rapid diagnosis plays an important role in early clinical management of patients due to lack of a vaccine and effective treatment. Laboratory diagnosis is generally accomplished by blood tests to detect virus-specific antibodies but these antibodies are usually developed one week after infection, which misses the window of effective clinical management. On the other hand, although detecting the viral genome can be done in early stage of infection by real-time polymerase chain reaction (PCR) but it is costly to the patients. Here we utilized a fluorescent compound to improve the cost-efficiency of the molecular assay for diagnosis of Chikungunya virus infection. By testing on 77 serum samples, this improved assay has proven to be highly sensitive and specific towards Chikungunya virus. We believe that this research could benefit both clinicians and patients by providing early and accurate diagnosis.

## Introduction

Chikungunya virus (CHIKV) is an arthropod-borne virus transmitted to humans primarily via the bite of an infected [1] *Aedes agypti* and *Aedes albopictus* mosquito. [2, 3] Currently, there are more than 40 countries including Africa, United States, European countries and Southeast Asian countries affected by chikungunya fever. [2] CHIKV is an enveloped positive-sense single stranded RNA virus belonging to *Alphavirus* genus of *Togaviridae* family. [4] The genome is approximately 11.8 Kb long, encoding four non-structural proteins (*nsP1*, *nsP2*, *nsP3*, *nsP4*) and five structural proteins (*C*, *E3*, *E2*, *6K* and *E1*). [5] The clinical symptoms of chikungunya fever are similar to that of dengue fever which is caused by Dengue virus (DENV), an arthropod virus belonging to *Flaviviriridae* family transmitted by same vectors as CHIKV. [6] This may result in cases of misdiagnosis in places where both viruses co-exist.

As there is no vaccine or specific therapeutic agent available for CHIKV infection, early diagnosis of CHIKV is crucial in preventing the collapse of health care system due to unprecedented number of cases usually encountered during CHIKV epidemics. [7] Virus isolation is classified as the gold standard in detection of CHIKV despite being a time-consuming process requiring 1–2 weeks to determine the presence of virus. The limitations associated with virus isolation resulted in the development of serological and molecular diagnostic methods that are rapid and less labour intensive. Enzyme-linked-immunosorbent assay (ELISA) and Immunochromatographic test (ICT) are examples of serological diagnostic assays which detect IgM and/or IgG antibodies that are specific to CHIKV present in patient sera. ELISA and ICT tests are inexpensive and easy to perform as they do not require handling live viruses. A four-fold increase in antibodies by comparing acute phase and convalescent phase serum samples is usually required to confirm CHIKV infection. IgM is detected on an average of two days after infection and persists for several weeks to three months, while IgG is detected in convalescent samples and may persist for years. [8] The outcome of having antibodies present in serum samples after recovery phase may deduce as false-positive detection. Blacksell and co-workers reported that commercially available antibody-based assays are not suitable for acute diagnosis of CHIKV as the results obtained showed ICT and ELISA kits having sensitivity of 1.9–3.9% and 3.9% respectively. [9] An alternative serological method of anti-CHIKV antibody detection has been reported to be used in commercial ELISA kits, but has shown cross-reactivity with other alphaviruses such as Ross River and O’ nyong-yong viruses as they are closely related serologically. [9] Thus, serological methods for CHIKV detection have been inefficient for acute phase diagnosis. [9–11]

Recently, molecular diagnosis has been well established for rapid, highly sensitive and specific detection of CHIKV infection during the acute phase. Viral RNA is extracted from serum samples collected 1–7 days post-infection [8] were detected by primers targeting the conserved regions of Chikungunya genome specifically. In comparison, conventional RT-PCR appears to be a less sensitive and relatively more time-consuming process than TaqMan and SYBR Green I-based real-time RT-PCR assays. However, real-time RT-PCR assays require highly sophisticated instruments with yearly maintenance and calibration, restricting the utilization of such assays in places with poor financial and technical resources. [12]

Previously, we have reported a novel diagnostic assay for CHIKV detection by adapting hairpin primers and fluorescent molecule, 2, 7-Diamino-1,8-naphthyridine (DANP), into a conventional PCR procedure. [13] In brief, DANP molecule contains a naphthyridine ring which enables it to bind specifically to a cytosine-bulge in a hairpin structure of the PCR primer by hydrogen bonds. [13] The binding of DANP molecule to DNA gives rise to a 400 nm excitation and 450 nm emission property to the bound DANP molecule. As PCR proceeds, the primer is incorporated into double stranded DNA and the hairpin is opened, causing the release of DNAP molecule and thereby decreasing the fluorescence intensity. [13] The utilization of DANP coupled hairpin PCR has also been demonstrated in a single-nucleotide polymorphism study of the cytochrome *P450* gene 2C9*3 by Takei and colleagues. [14] However, the binding of DANP molecule to the hairpin-primer is in an equilibrium manner, so that excess DANP molecules must be added to ensure a detectable fluorescence intensity. Therefore, the background signal given off by unbound DANP molecules limits the sensitivity and consistency of the assay.

**Fig 1:**
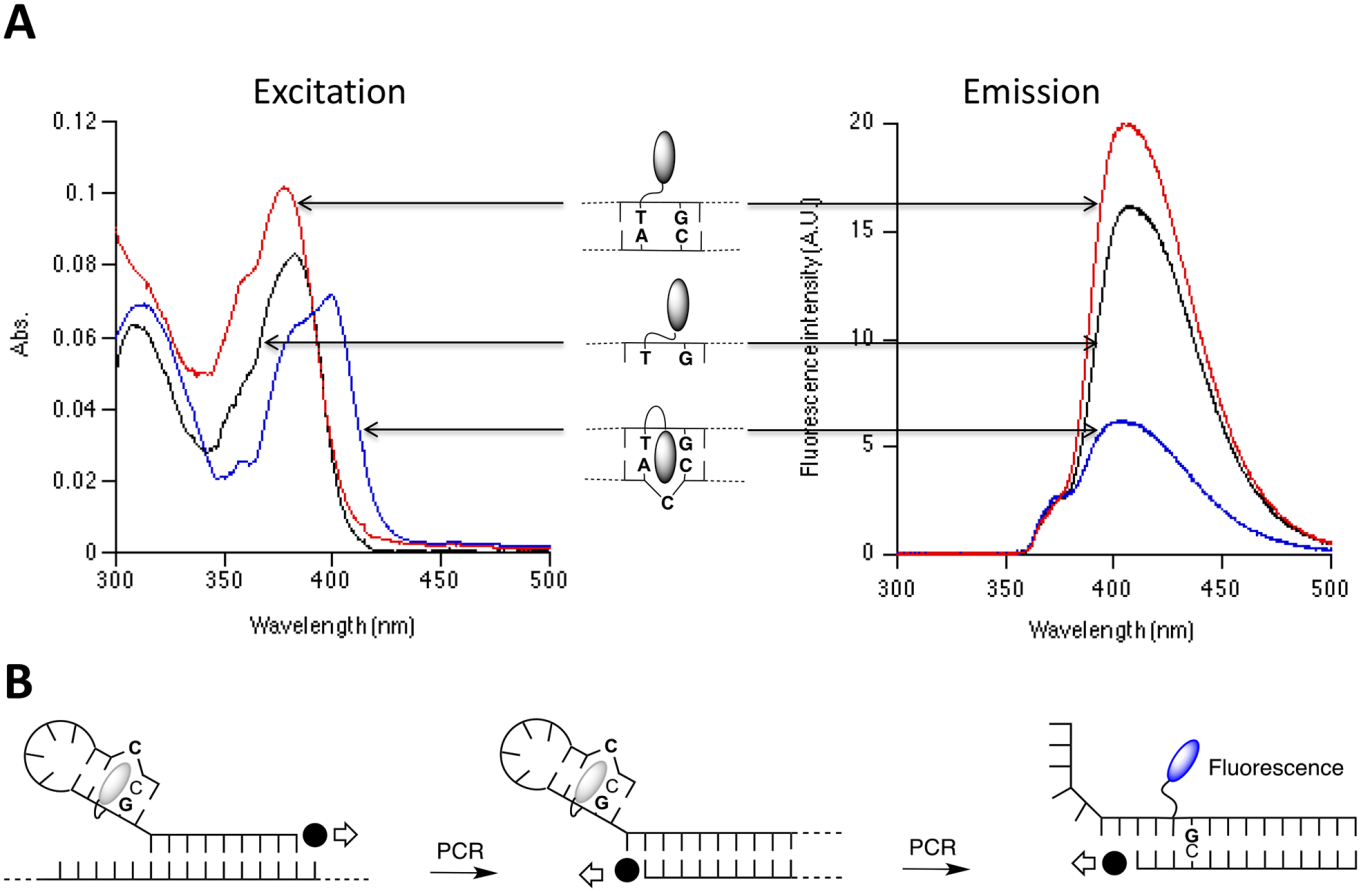
A. Excitation-emission spectrum of DANP-DNA complexes. 365 nm UV-light is selected for excitation because only basal level of absorbance by DANP-C-bulge complex can be seen while the DANP-dsDNA complex absorbed substantially at this wavelength. Similarly, emission light at 430 nm is measured mainly because it generates the most significant difference when DANP-dsDNA and DANP-C-bulge complexes. B. Illustration of the chemical binding change happens to DANP molecule during PCR procedure.

In the present study, DANP molecule was covalently immobilized on the hairpin PCR primers containing C-G base-pairs directly after the C-bulge to quench the fluorescence emission, as shown in Fig 1A. As PCR progresses, the hairpin structure is opened up and the DANP molecule is moved to the outer surface of the double-stranded DNA molecule, away from cytosine-bulge, resulting in an increase in fluorescence emission at 430 nm when it is subjected to UV-light at 365 nm. Increments in fluorescence intensity can be picked up only if the viral RNA template is present in the reaction with negligible background signal as no excess DANP molecules were added to the reaction. The method is highly effective as it uses a conventional RT-PCR protocol followed by measurement of fluorescence signal intensity using a spectrophotometer. The assay is more rapid and cost-effective as compared to real-time PCR methods. The assay was also validated with CHIKV infected patient serum samples and healthy individual serum samples for its sensitivity and specificity.

## Materials and Methods

### Viruses

CHIKV (GenBank accession No. FJ445502) was isolated from an infected patient during the CHIKV outbreak in Singapore in 2008. The virus was propagated in *Aedes albopictus* C6/36 cells. Briefly, cells were grown to about 80% confluency in T75 tissue culture flasks. Following removal of the growth media, virus inoculum was added to give a multiplicity of infection (MOI) of 0.1 PFU/cell. Flasks were incubated at 28°C for 1 hours with constant agitation at every 15 min interval. After the incubation, Rosewell Park Memorial Institute (RPMI) 1640 growth medium (Sigma-Aldrich Corp) supplemented with 2% FBS (Hyclone) was added and flasks were maintained at 28°C for about 3–5 days or until cells showed 80% cytophatic effects (CPE). The viral titers were determined by plaque forming assay. [13] Ross River virus (RRV), Sindbis virus (SINV), Kunjin virus (KUNV, MRM 61C strain), West Nile virus (WNV, Sarafend strain), Zika virus (ZIKV, MR 766 strain), DENV-1 (S144 strain), DENV-2 (New Guinea C strain), DENV-3 (Eden 130/05 strain), DENV-4 (S8976 strain), Influenza A virus subtype H1N1, H3N2, Poliovirus type 1 (PV1, Sabin strain), type 2 (PV2, Sabin strain), type 3 (PV3, Sabin strain), Human enterovirus 71 (HEV71, AF316321 strain), Coxsackie B2 virus (CB2), Coxsackie A16 virus (CA16, WHO strain) and Enteric cytopathic human orphan virus 7 (Echo7) were also used to examine the cross-reactivity of this assay. The ZIKV, DENV1-4, PV1-3, HEV71, CB2, CA16 and Echo7 viruses were maintained in the laboratory. The RRV, KUNV and WNV were kindly provided by Professor Mary Mah-Lee Ng, Department of Microbiology, National University of Singapore. The Influenza A viruses were kindly provided by Associate Professor Tan Yee Joo, Department of Microbiology, National University of Singapore.

### Ethics statement

A set of 22 serum samples from CHIKV-infected patients, and 30 from uninfected individuals were collected at the National University Hospital, Singapore, with informed consent, to evaluate the clinical sensitivity and specificity of the DANP-anchored assay. All of the sera were confirmed as febrile illness associated with a positive result from the real-time RT-PCR. [15] This part of the study was performed in accordance with the National University of Singapore Institutional Review Board approved protocol (No. 10–234). Environmental Health Institute (EHI), National Environmental Agency of Singapore kindly provided a set of 25 serum samples from clinically-suspected patients in which the presence of CHIKV was confirmed by a real-time RT-PCR assay. [16] Written informed consent was given for all samples involved in this study.

### Viral RNA extraction

CHIKV RNA was extracted from 140 μL of infected cell culture supernatants (3.6 X 10^^7^ PFU/mL) and serum samples using the QIAamp viral RNA mini kit (Qiagen, Hilden, Germany) according to the manufacturer’s instructions. The RNA was eluted in a final volume of 50 μL of nuclease-free water and stored at −80°C until use.

### PCR Primer designing

The full genomes of multiple geographically different strains of CHIKV from recent outbreaks were retrieved from GenBank and aligned using the ClustalX (version 2.1) [17] sequence alignment software. Primers were designed to target the highly-conserved *nsP2* regions of CHIKV genome, as shown in Table 1. The primers were designed with hairpin (underlined sequences in Table 1) at the 5’ end to accommodate DANP molecule which is covalently conjugated to the thymine nucleotide (bolded sequences in Table 1) of the primers.

**Table 1:** DANP-anchored hairpin primer sequences used for detection of CHIKV.

Primers	Sequence
DANP-ANCHORED F	5’ ATCA**T**GCTTTTGCCATGATGACTAATCCGCCCTACCACG 3’
DANP-ANCHORED R	5’ ATCA**T**GCTTTTGCCATGATGCATCCATTCAAGAGCAGCG 3’

### RT-PCR conditions

RT-PCR reactions were performed in C1000 thermal cycler (Bio-Rad, Hercules, CA). Reactions were optimized with a One Step RT-PCR kit (Biotech Rabbit, Hannover, Germany). Each reaction was performed in 25 μL total reaction mixture containing 12.5 μL of 2x reaction buffer, 0.2 μmol/L of each forward and reverse DANP hairpin primers, 1.25 μL of 20x RT-RI blend (reverse transcriptase and RNAse Inhibitor) and 1 μL of viral RNA. 10 μL from each total reaction volume was set aside while the remaining 15 uL of reaction was subjected to RT-PCR. The thermal profile was optimized as follows; reverse transcription step at 45°C for 20 minutes, activation of *Taq* polymerase at 95°C for 2 minutes, followed by 30 cycles of PCR cycling steps consisting of 95°C for 10 seconds, 60°C for 10 seconds and 72°C for 15 seconds.

### Fluorescence intensity measurement and native PAGE analysis

In order to determine the fluorescence intensity, 10 μL of each reaction was diluted with 90 μL of nuclease-free water in each well of a white opaque flat-bottom 96-well plate. The fluorescence intensity from each well was scanned by Infinite^®^ 200 PRO microplate reader (Tecan Trading AG, Switzerland) with an excitation filter at 365-nm and an emission filter at 430-nm. A sample positive for CHIKV infection was determined as the increment in fluorescence intensity after PCR was more than 100 arbitrary units (AU) as compared to background fluorescence in pre-PCR reaction mixture. For assay validation, all PCR products were analysed using 8% native polyacrylamide gel electrophoresis (PAGE), followed by ethidium bromide staining for two minutes. Gel images were captured using the GeneSnap software version 7.02 (Syngene, Cambridge, UK).

### PCR cycle number optimization

In order to determine the number of PCR cycles that gives off the most significant increment in fluorescence intensity after PCR, the fluorescence intensity level was measured and compared after every five PCR cycles using CHIKV genomic RNA as positive control and nuclease-free water as negative control (NTC).

### Determination of limit of detection of the assay

CHIKV RNA was extracted from 140 μL of infected cell culture supernatants with viral titre of 3.6 X 10^^7^ PFU/mL, using the QIAamp viral RNA mini kit (Qiagen, Hilden, Germany) according to the manufacturer’s instructions. The viral RNA was eluted in a final volume of 50 μL of nuclease-free water and then serial diluted logarithmically until a final concentration of 1 X 10^−5^ PFU/μL. 1 μL of each of the serial diluted viral RNA samples was subjected to the DANP-anchored RT-PCR assay to determine the limit of detection of the assay.

The RNA concentration ranges tested were 1.0 X 10^1^ to 1.0 X 10^−5^ PFU/reaction (3.6 X 10^3^ to 3.6 X 10^−3^ PFU/mL).

### Cross reactivity study

RRV, SINV, KUNV, WNV, ZIKV, DENV1-4, Influenza H1N1, H3N2, PV1-3, HEV71, CB2, CA16 and Echo7 were used to examine the cross-reactivity of this assay. Viral RNA was extracted from 140 μL of each of the viruses and eluted in 50 μL of nuclease-free water. The RNA concentration were measured using NanoDrop ND2000 Spectrophotometer (NanoDrop Technologies, Inc., Wilmington, DE, USA) and only samples with at least 50 ng/μL were proceeded to cross reactivity study of the assay.

## Results

### PCR cycle optimization

**Fig 2:**
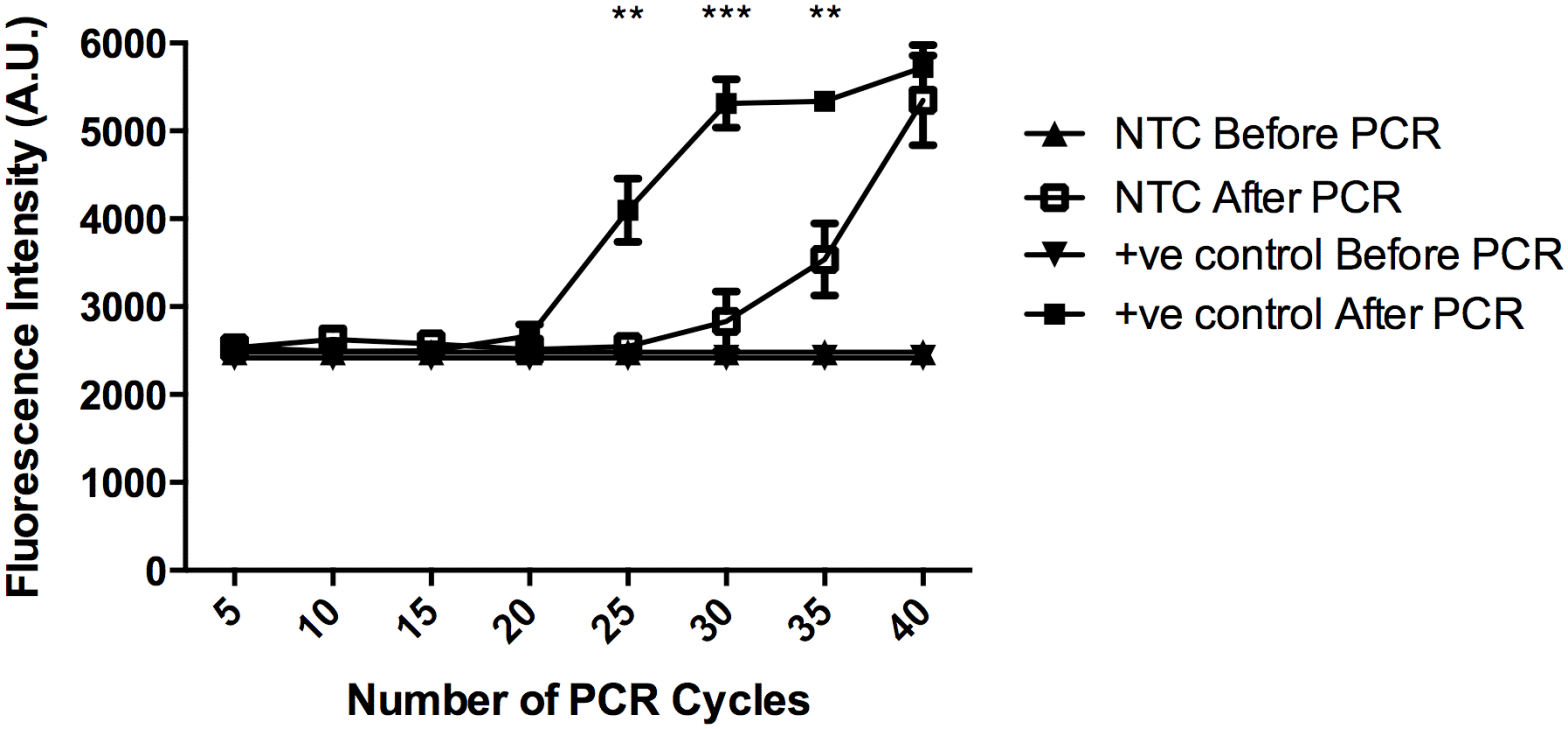
Optimization of number of PCR cycles. DANP-anchored RT-PCR is carried out with and without the presence of CHIKV RNA template and the fluorescence intensity is measured after every 5 PCR cycles from both before and after PCR reactions. The fluorescence intensity starts to increase significantly after 20 cycles when CHIKV RNA is present and reaches saturation after 30 cycles, while that of NTC also starts to increase slowly from 25 to 30 cycles and become more obvious afterwards. As a result, the maximum difference in fluorescence intensity can be achieved after 30 cycles of PCR reaction. Data are shown as means SEM of five experiments. ***P < 0.001, **P < 0.01 by multiple t-test.

In order to determine the number of PCR cycles that gives off the most significant increment in fluorescence intensity after PCR, the fluorescence intensity level was measured and compared after every five PCR cycles using CHIKV genomic RNA as positive control and nuclease-free water as negative control (NTC). As shown in Fig 2, the difference in fluorescence intensity between before PCR and after PCR samples reached the maximum at 30 cycles. Due to the formation of primer dimer and non-specific PCR products in the NTC, the difference in fluorescence intensity began to narrow down after 30 cycles. Therefore, 30 cycles of PCR reaction was used in the rest of the study.

### Primer validation

**Fig 3:**
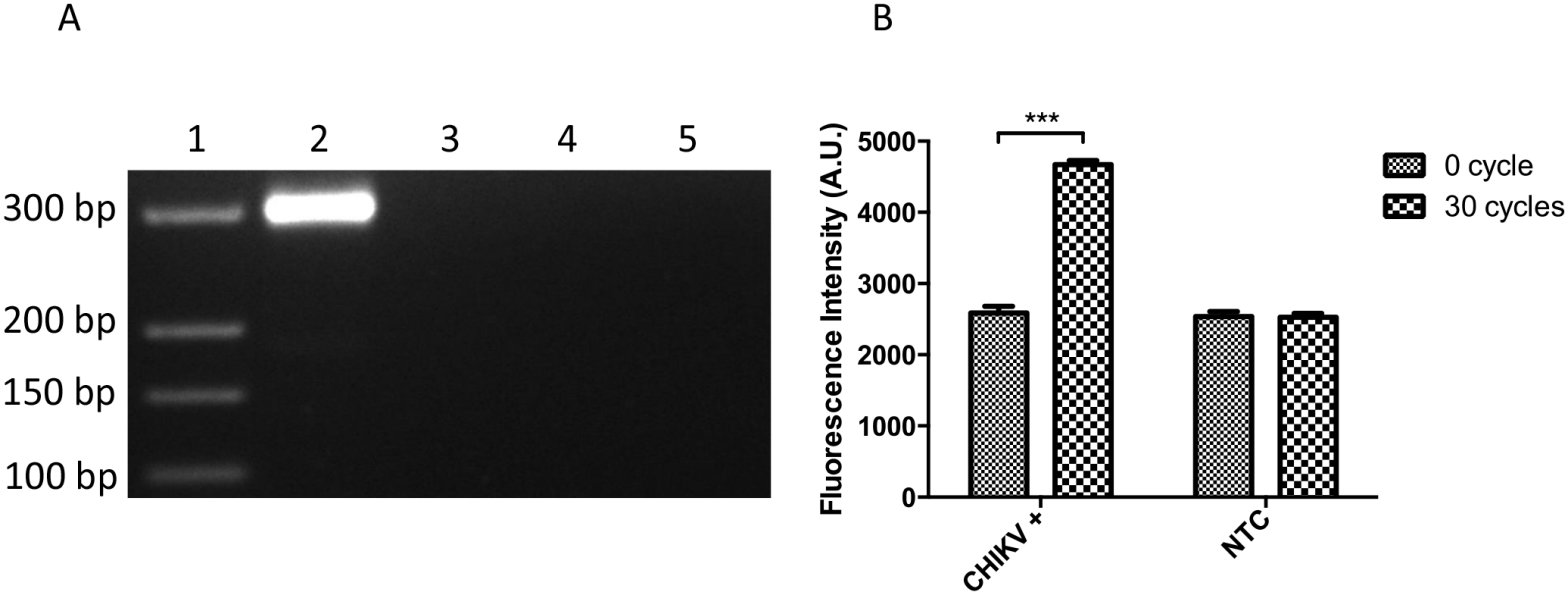
DANP-anchored RT-PCR primer was evaluate. A: Specific PCR product with expected size (296 bp) is observed only in reaction with CHIKV RNA template after 30 PCR cycles. Lane 1, DNA ladder (GeneRuler Ultra Low Range, Thermal Fisher Scientific, Waltham, Massachusetts, USA); lane 2, CHIKV RNA+ after 30 PCR cycles; lane 3, CHIKV RNA+ before PCR; lane 4, NTC after 30 PCR cycles; and lane 5, NTC before PCR. B: The fluorescence intensity from the PCR reactions with and without CHIKV RNA before and after 30 PCR cycles. Only the reaction with CHIKV RNA shows significant increment in fluorescence intensity (approximately 2000 AU) after 30 cycles of PCR reaction. Data are shown as means SEM of five experiments. ***P < 0.001 by Student’s t-test.

To validate the suitability of hairpin primers and to verify the initial fluorescence intensity level, DANP-anchored hairpin RT-PCR procedure was carried out with and without CHIKV genomic RNA. All PCR products were analyzed by PAGE to determine the assay specificity. As indicated in Fig 3A, the specific PCR product of 296 bps can only be seen when CHIKV RNA is present. There was no significant change in fluorescence intensity between before/after PCR in NTC reactions (Fig 3B). In contrast, an increment of more than 2000 AU of fluorescence intensity was observed after 30 cycles of PCR in the presence of CHIKV RNA.

### Detection limit of the assay

**Fig 4:**
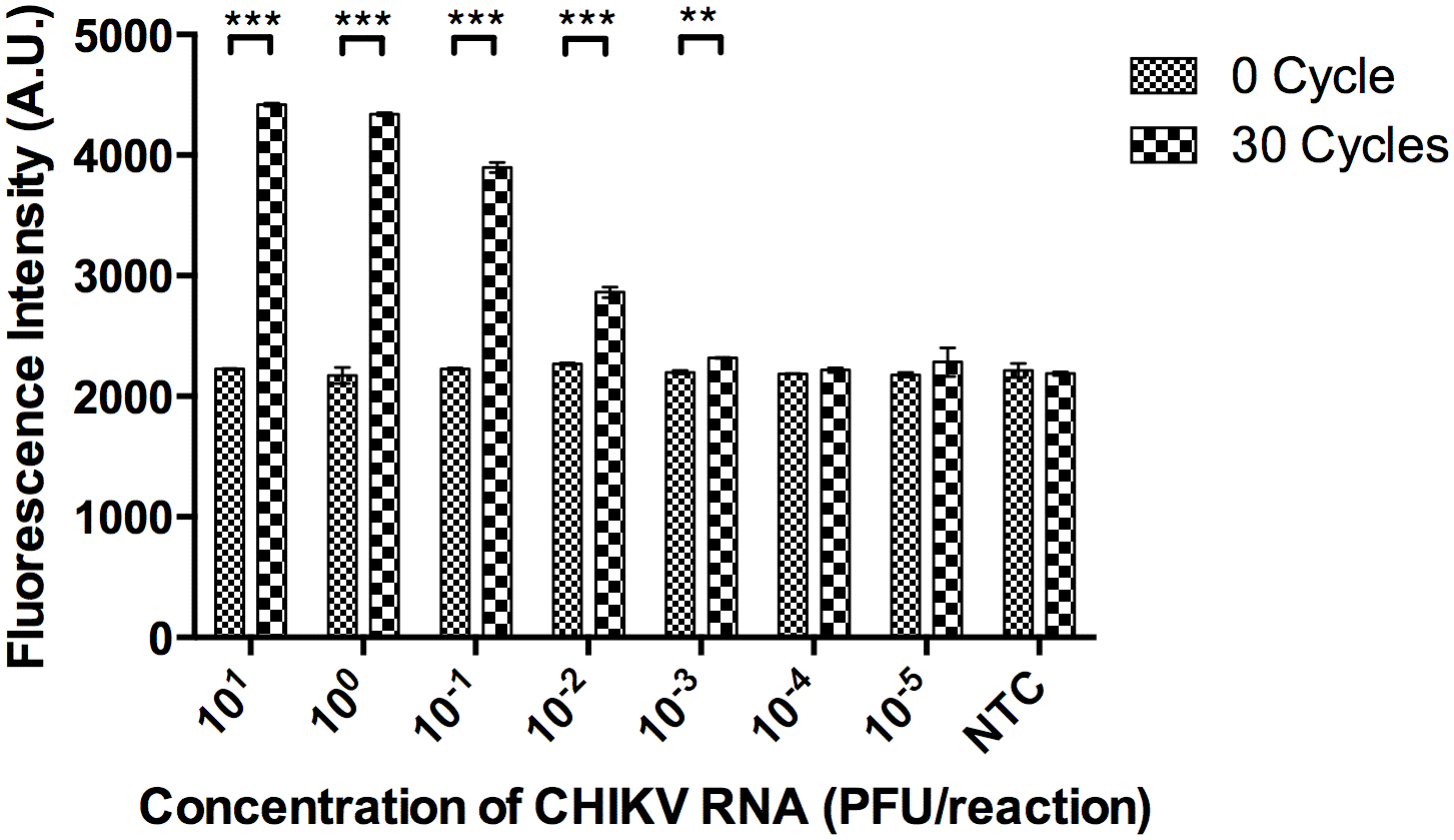
The detection limit of a DANP-anchored RT-PCR assay was determined using 10-fold serial diluted CHIKV genomic RNA. The detection limit for the assay was equivalent to 0.001 PFU per reaction of CHIKV. Data are shown as means SEM of three experiments. ***P < 0.001, **P < 0.01 by Student’s t-test.

The detection limit of the DANP-anchored RT-PCR assay was determined through replicates of reactions, using serial logarithmic dilutions of the control CHIKV genomic RNA. Fig 4 shows the change in fluorescence intensity before and after 30 cycles of PCR reaction. A statistically significant increase of 120 AU was observed in 0.001 PFU per reaction, the lowest level of detection by the assay.

### Cross-reactivity of the assay

**Fig 5:**
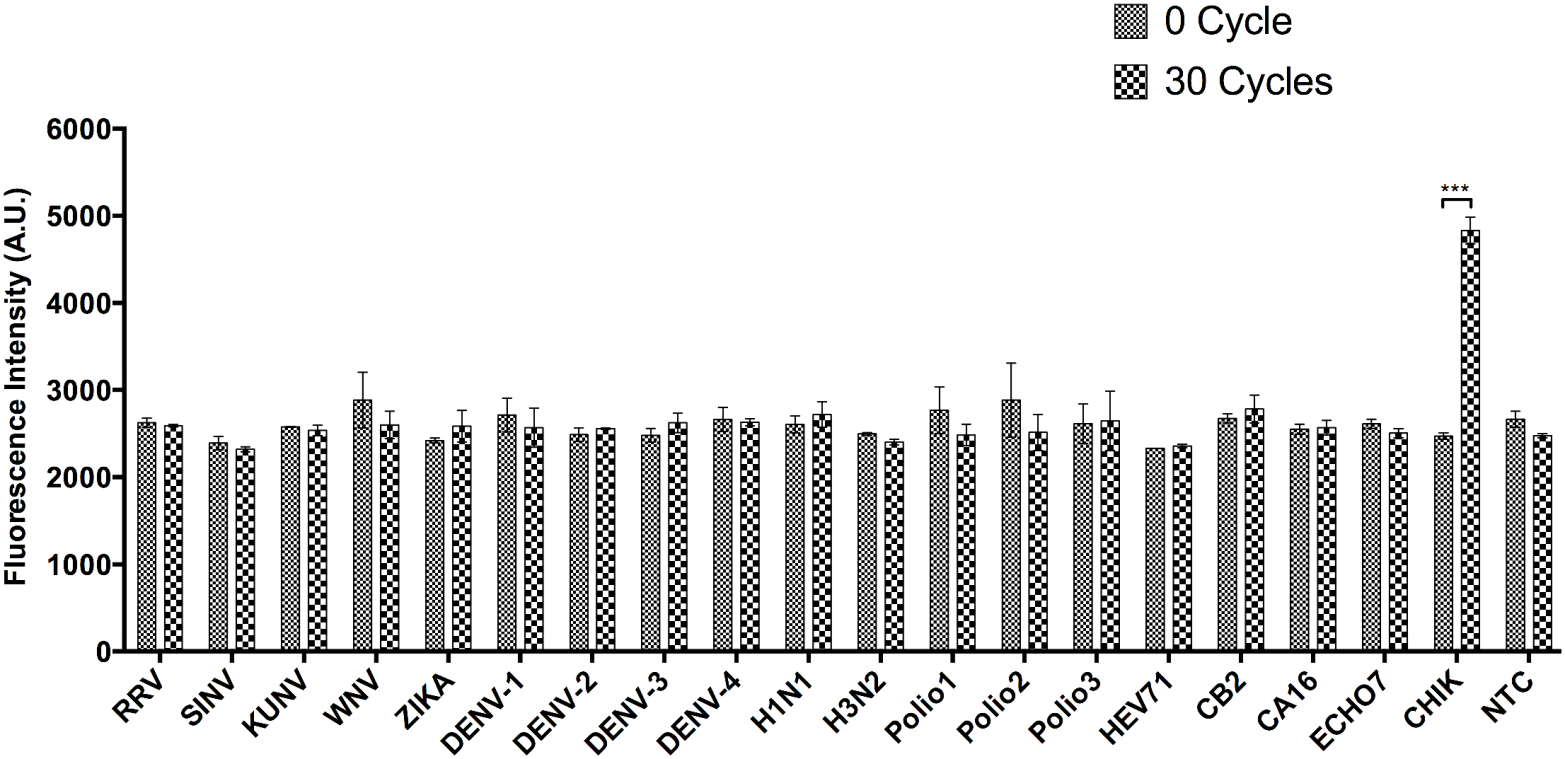
Cross-reactivity of DANP-anchored RT-PCR was evaluated with a panel of positive-sense RNA viruses. There is no significant increase in fluorescence intensity found in any one of the viruses tested showing no cross-reactivity of the assay to these viruses. Data are shown as means SEM of three experiments. ***P < 0.001 by Student’s t-test.

The cross-reactivity of the assay was determined by using a panel of other RNA viruses. RRV and SINV were used as representative members of *Alphavirus*; KUNV, WNV, ZIKV and DENV-1, DENV-2, DENV-3, and DENV-4 were used as representative members of *Flavivirus* in the cross-reactivity study. The remaining RNA viruses included H1N1, H3N2, Polio 1, Polio 2, Polio 3, HEV71, CB2, CA16 and ECHO7. Only CHIKV RNA samples demonstrated positive results, indicating the lack of cross-reactivity with other viruses tested (Fig 5).

### Sensitivity and specificity of the assay

In order to evaluate the sensitivity and specificity of the present assay for clinical diagnosis, 47 serum samples obtained from patients with confirmed CHIKV infection during the acute phase and 30 serum samples from uninfected individuals were tested. The present assay demonstrated high sensitivity by picking up 44 of the 47 CHIKV cases (93.62% sensitivity; 95% CI, 81.44% to 98.37%). None of the 30 serum samples from uninfected individuals was false diagnosed as positive (100% detection specificity; 95% CI, 85.87% to 100%) (Table 2).

**Table 2:** Performance of DANP-ANCHORED on Serum samples.

Type of Serum Sample	No. of samples	No. of samples diagnosed as positive	Sensitivity^†^	Specificity^‡^
CHIKV	47	44	93.62% (95% CI, 81.44% to 98.37%).	100% (95% CI, 85.87% to 100%)
Healthy	30	0		

^†^ Number of positive specimens/(number of positive specimens + number of false-negative specimens) X 100%.

^‡^ Number of negative specimens/(number of negative specimens + number of false-positive specimens) X 100%.

## Discussion

CHIKV has been relatively understudied as it was restricted to Africa and Asia countries. [18] Since 2005, CHIKV started to spread to countries in Indian Ocean and then globally. [19] It is estimated that > 1.5 million people were infected in India during the 2006 outbreak alone, [20] and, currently, Chikungunya fever has been documented in more than 40 countries. [21] The unprecedented worldwide spread of CHIKV was driven by international travel and the A226V mutation on the *envelope protein 1*, which better adapts the virus to *Aedes albopictus*. [3] Due to increased globalization and mosquito vectors expand to new areas, early diagnosis of CHIKV is critical in the absence of any licensed antiviral therapy and prophylaxis, especially in developing countries.

Currently, the diagnosis of CHIKV largely relies on virus isolation, detection of specific antibody and nucleic acid. Virus isolation in tissue culture is time-consuming and technically complex that is limited in developing countries. Because of extensive cross-reaction between *alphaviruses* due to common antigens, serological assays often face the difficulty in differentiating commonly occurring *alphaviruses*. These drawbacks have made molecular assays the method of choice for diagnosis during acute phase of chikungunya fever. Molecular techniques based on the detection of genomic sequences by RT-PCR, nested RT-PCR, and real-time RT-PCR are rapid and sensitive and have replaced virus isolation as the new standard method for the detection of CHIKV in acute-phase serum samples, but the reagents and equipment are too costly for widespread use. In this regard, this DANP-anchored RT-PCR assay reported in this study is advantageous, because of its simplicity, rapidity, and cost-effectiveness. Only a standard conventional PCR procedure, with DANP hairpin primer used, and a fluorescence reading procedure is required without involving of sophisticated instrument or costly reagent.

In comparison, previously, we have reported a novel DANP-coupled hairpin RT-PCR for rapid detection of CHIKV in the acute phase serum samples. PCR primers were designed specifically to target *nsP2* gene of CHIKV with hairpin tag containing a cytosine-bulge. The DANP molecule binds to the C-bulge in its protonated form (DANPH+) before PCR reaction starts, resulting in fluorescence emission. During PCR amplification, the hairpin primer opens up and releases the DANP molecule resulting in a drop in fluorescence emission giving rise to a ‘turn-off’ system. CHIKV positive samples are determined by comparing the fluorescence intensity recorded before and after PCR process by subjecting the PCR products to UV-light and detecting the emitted fluorescence at 430 nm. Despite it was a rapid, sensitive, specific and cost effective assay; the optimization of DANP concentration due to background signal restricted its usage. In order to overcome the issue, in the present study, we covalently conjugated the DANP molecule onto the hairpin structure of the PCR primer. Therefore, the ratio between DANP molecule and primer is fixed at 1:1 and this standardization simplifies the optimization of the assay. In addition, by changing the reading spectrum from 400 nm excitation and 450 nm emission to 365 nm excitation and 430 nm emission, we managed not only to minimize the background signal but also give rise to a ‘turn-on’ system. In addition, we have also shortened the assay turnaround time from 90 minutes to 60 minutes including fluorescence reading, by cutting down the reverse transcription step duration and optimizing the PCR cycle number from 40 to 30.

The detection limit of the assay was 0.001 PFU per reaction that is lower than that of the previous DANP coupled assay [13] and is comparable to real time RT-PCR assays developed by other groups [22, 23]. A side-by-side comparison of our assay with the *ab*TES DEN 5 qPCR I Kit (Cat: 300152) from AIT biotech, a Taqman probe-based multiplex real time RT-PCR for DENV/CHIKV detection. Comparable limit of detection was noted. Despite the fact that the viremia load is usually above 4 log_10_ during the acute phase of CHIKV infection, [24] low detection limit of the assay enables us to detect CHIKV RNA even during late acute phase when the viral titers start to decline rapidly. More importantly, the present assay is not cross-reactive with a panel of RNA viruses that are co-circulating in endemic regions. Given that CHIKV is commonly misdiagnosed as DENV and vice versa, the outstanding specificity of our assay could benefit both clinicians and patients at the point-of-care by providing accurate diagnosis.
